# The Rice DNA-Binding Protein ZBED Controls Stress Regulators and Maintains Disease Resistance After a Mild Drought

**DOI:** 10.3389/fpls.2020.01265

**Published:** 2020-08-18

**Authors:** A. Paola Zuluaga, Przemyslaw Bidzinski, Emilie Chanclud, Aurelie Ducasse, Bastien Cayrol, Michael Gomez Selvaraj, Manabu Ishitani, Alain Jauneau, Laurent Deslandes, Thomas Kroj, Corinne Michel, Boris Szurek, Ralf Koebnik, Jean-Benoit Morel

**Affiliations:** ^1^BGPI, INRA, CIRAD, SupAgro, Univ. Montpellier, Montpellier, France; ^2^Valle del Cauca, CIAT, Palmira, Colombia; ^3^Institut Fédératif de Recherche 3450, Université de Toulouse, CNRS, UPS, Plateforme Imagerie TRI-Genotoul, Castanet-Tolosan, France; ^4^LIPM, Université de Toulouse, INRA, CNRS, Castanet-Tolosan, France; ^5^UMR Interactions Plantes-Microorganismes-Environnement (IPME), IRD-Cirad-Université Montpellier, Institut de Recherche pour le Développement, Montpellier, France

**Keywords:** Zn-finger BED domains, *Magnaporthe oryzae*, disease resistance, drought resistance, plant–pathogen interactions

## Abstract

**Background:**

Identifying new sources of disease resistance and the corresponding underlying resistance mechanisms remains very challenging, particularly in Monocots. Moreover, the modification of most disease resistance pathways made so far is detrimental to tolerance to abiotic stresses such as drought. This is largely due to negative cross-talks between disease resistance and abiotic stress tolerance signaling pathways. We have previously described the role of the rice ZBED protein containing three Zn-finger BED domains in disease resistance against the fungal pathogen *Magnaporthe oryzae*. The molecular and biological functions of such BED domains in plant proteins remain elusive.

**Results:**

Using *Nicotiana benthamiana* as a heterologous system, we show that ZBED localizes in the nucleus, binds DNA, and triggers basal immunity. These activities require conserved cysteine residues of the Zn-finger BED domains that are involved in DNA binding. Interestingly, ZBED overexpressor rice lines show increased drought tolerance. More importantly, the disease resistance response conferred by ZBED is not compromised by drought-induced stress.

**Conclusions:**

Together our data indicate that ZBED might represent a new type of transcriptional regulator playing simultaneously a positive role in both disease resistance and drought tolerance. We demonstrate that it is possible to provide disease resistance and drought resistance simultaneously.

## Background

In agriculture, biotic and abiotic stresses cause important yield losses, making improvement in stress tolerance of crop plants a major aim for research. Rice (*Oryza sativa*) has a pivotal role for the food security of over half the world’s population (Calpe, 2006). Among biotic stresses, rice blast caused by the fungal pathogen *Magnaporthe oryzae* is arguably one of the most devastating diseases in rice because of its wide distribution and the potential of causing total losses under conducive conditions (Hyun-Khang and Valent, 2010). Additionally, under rainfed cultivation, rice is one of the most drought-susceptible crops because of its small root system, thin cuticular wax, and rapid stomata closure (Oladosu et al., 2019). Plants have developed regulatory networks allowing them to adapt, survive, and reproduce under several stress conditions (Kissoudis et al., 2014). Two major types of resistance against pathogens are often defined according to the molecular mechanism for pathogen recognition (Jones and Dangl, 2006). The first layer of active defense is triggered by highly conserved pathogen-associated molecular patterns (PAMPs). PAMP-triggered immunity (PTI), also called basal immunity, confers basal disease resistance following infection by a virulent pathogen. The other layer of defense, called effector-triggered immunity, is activated by direct or indirect recognition of effector molecules from the pathogen by resistance proteins of the plant and is leading to programmed cell death, preventing the spread of the pathogen in the host (Jones and Dangl, 2006). In both forms of resistance several physiological changes occur, including the generation of reactive oxygen species (ROS), ion fluxes, accumulation of defense hormones such as salicylic acid (SA), and induction of defense-related genes, such as pathogenesis-related (*PR*) genes (Corwin and Kliebenstein, 2017).

The main components of these regulatory networks are shared by abiotic stress as well, including ROS signaling, ion fluxes, and plant hormone signaling among others (Zhang and Sonnewald, 2017). However, negative cross-talks between disease resistance and abiotic tolerance pathways often render pathogen-resistant plants into abiotic-sensitive plants and the other way around (Kissoudis et al., 2014; Suzuki et al., 2014). For instance, the phytohormone abscisic acid (ABA), one of the main regulators of drought-stress response is also known to alter the plant response against pathogens by negatively regulating both salicylic acid (SA) and jasmonic acid (JA) biosynthesis, and signaling (Kissoudis et al., 2014; Ramegowda and Senthil-Kumar, 2015). Several studies of transgenic plants have shed light into some of the mechanisms by which rice copes with different stresses. For instance, the ERF family transcription factor OsLG3 rice protein increases tolerance against drought by scavenging reactive oxygen species (ROS) (Xiong et al., 2018). The stress response mitogen-activated protein kinase (MAPK) from rice OsMAPK5 positively regulates drought, salt, and cold tolerance but negatively modulates disease resistance against the rice blast fungus *Magnaporthe oryzae* and the bacterial pathogens *Burkholderia* glumae (Xiong and Yang, 2003) or *Xanthomonas oryzae* (*Xoo*) (Seo et al., 2011). Similarly, overexpression of *NPR1*, a gene acting as a positive regulator of disease resistance, leads to reduced tolerance to drought (Quilis et al., 2008). Out of 60 cases of transgenic rice plants showing enhanced disease resistance reviewed, 28 showed detrimental effects for other stress or developmental defects (Delteil et al., 2010). Conversely, there are few examples where the enhancement of disease resistance leads to an improved tolerance to abiotic stress. For instance, the calcium-dependent protein kinases OsCPK4 and OsCPK10 as well as the transcription factor *OsNAC6* positively enhance tolerance against drought, salinity as well as resistance towards *M. oryzae* (Nakashima et al., 2007; Campo et al., 2014; Bundó and Coca, 2016; Bundó and Coca, 2017). Similarly, OsWRKY11 (Lee et al., 2018) and MAPK kinase 10.2 (Ma et al., 2017) enhance resistance against *Xoo* and drought. Additionally, the MADS-box transcription factor OsMADS26 negatively regulates resistance against *M. oryzae*, *Xoo* and drought in rice (Khong et al., 2015). These non-exhaustive examples demonstrate the complexity of the connections between plant response pathways to abiotic and biotic stresses.

The response of plants to combined stress, in particular combination of biotic and abiotic stresses, revealed to be even more complex than the response to single stresses (for review Zhang and Sonnewald, 2017). In the case of combined disease and drought stresses, the outcome of the interaction leading to either susceptibility or resistance is influenced by various factors including the nature of the pathogen, the developmental stage of the plant, as well as the severity and the duration of the drought stress (Ramegowda and Senthil-Kumar, 2015; Zhang and Sonnewald, 2017). For example, exposure to mild drought induces basal defenses (Ramegowda and Senthil-Kumar, 2015), while severe drought conditions reduce basal defenses and inhibit disease resistance mediated by resistance genes (Bidzinski et al., 2016). Thus, identifying genetic determinants conferring a disease resistance response that remains effective under abiotic stress like drought is an important challenge for research.

We previously identified a rice protein containing three zinc-finger BED domains called ZBED, which was shown to be a positive regulator of resistance against the rice blast fungus (Kroj et al., 2016). The BED-type zinc finger (zf-BED) domain is a protein domain that was named after the Drosophila BEAF (**B**oundary **E**lement-associated factor) protein, thought to be involved in chromatin insulation and the Drosophila **D**REF protein, a transcriptional regulator for S-phase genes (Aravind, 2000). The BED finger is about 50 to 60 amino acid residues domain that contains a characteristic motif with two highly conserved aromatic positions, as well as a shared pattern of cysteines and histidines, predicted to form a zinc finger (zf). It has been suggested that DNA-binding is the general function of this domain. Some proteins known to contain BED domains are the Hobo-like transposases (Aravind, 2000); *Caenorhabditis elegans* Dpy-20 protein, a predicted cuticular gene transcriptional regulator (Inoue and Sternberg, 2010); and tobacco 3AF1 (Lam et al., 1990) and tomato E4/E8-BP1, light- and ethylene-regulated DNA-binding proteins (Coupe and Deikman, 1997). BED domains were also found to be integrated into plant resistance genes from different plant species like poplar (*Populus trichocarpa*) and in several monocot species such as *Oryza sativa*, *Hordeum vulgare*, *Brachypodium distachyon* and *Triticum* spp among others (Yoshimura et al., 1998; Kroj et al., 2016; Marchal et al., 2018; Read et al., 2020). However, the role of the zf-BED domain in plant resistance genes has not been elucidated. We proposed that such integrated BED domains could represent decoys of endogenous host BED proteins targeted by pathogen effectors (Read et al., 2019). However, the recent finding that the Yr7, Yr5, and YrSP resistance genes from wheat have identical zf-BED sequences as Xa1, CGS-Xo111, and Nb-xo15 from rice (Marchal et al., 2018; Read et al., 2019), suggests that this domain is not a specificity-determining decoy. Rather, it may have a role in downstream signaling, localization, or other. The way BED domains operate might include dimerization, recruitment of other interacting proteins, or DNA binding. Thus, the molecular and biological functions of BED-containing proteins in plants are still largely unknown. Given the multiple cases of negative cross-talks already mentioned, we wanted to better understand the potential role of ZBED in disease resistance by testing bacterial pathogens, and to assess whether ZBED has a role in abiotic stress response in rice. This led us to test if disease resistance conferred by ZBED is robust under drought.

## Materials and Methods

### Plant Genotypes

Rice plants (*Oryzae sativa*) Nipponbare background were used for Agrobacterium transformation to generate transgenic lines overexpressing the *ZBED* gene (LOC_Os01g36670) as described (Kroj et al., 2016). Briefly, the ZBED cDNA from rice cv Nipponbare (L.) was PCR amplified and cloned into the pBIOS2300OX transformation vector under the control of the constitutive maize (*Zea mays*) ubiquitin promoter and plants were transformed as described previously (Yuan et al., 2007). The number of T‐DNA insertions was estimated in the T1 and T2 families using PCR for the geneticyn/kanamycin selection marker as a diagnostic for the presence of T-DNA. Lines carrying single copy insertions (showing 3:1 PCR-positive; *n *>* *20 plants analyzed) were conserved for further analysis. Siblings PCR-negative (not containing the transgene) were used as controls (azygous controls). Individual T2 plants (homozygous overexpressor and azygous) from three independent monolocus lines were selfed, and *ZBED* overexpression was confirmed in the T3 progeny (Grand et al., 2012). Three overexpressing *ZBED* lines (ZBED-OX1, ZBED-OX2, and ZBED-OX3) with their respective azygous lines (ZBED 1/0, ZBED 2/0, and ZBED 3/0) were used in this study.

### Yeast Two-Hybrid Screening (Y2H)

For Y2H, we used the library (in the Co39 Indica background) and methods described in (Cesari et al., 2013). We also built a new library (RCL1) from japonica rice Nipponbare using RNA from leaves and shoot-apical meristem of flowering plants, after drought stress (root and leaves treated by Mannitol or salt) and disease-related stresses (leaves treated with blast fungus or chitin for one and two days). Both libraries were made in the Matchmaker system (Clontech) following the manufacturer’s instructions. Once identified, full-length cDNAs of ZBED interactors (KIP1, WRKY4, STE20 and STE11) were cloned and further used for BiFC analysis.

### Bimolecular Fluorescence Complementation (BiFC)

Full-length cDNAs were cloned into the pDONR207 (Thermo Fischer Scientific). The N-terminal portion of yellow fluorescent protein (YFPn) was fused to the N-terminus of KIP1, STE20, STE11 and WRKY4. Each of those constructs was co-expressed in *N. benthamiana* leaves with YFPc-ZBED (carboxy-portion of YFP fused to the N-terminus of ZBED). Each construct was infiltrated in three *N. benthamiana* leaves (technical reps), and the experiment was repeated twice for a total of three biological reps. The YFP fluorescence complementation was analyzed by laser-scanning confocal microscope, laser power was set at 2% for all interactions, except WRKY4 which was set at 4%. The nuclei were DAPI-stained.

### Generation of Point Mutations in ZBED’s BED Domains

The ZBED (LOC_Os01g36670) cDNA, cloned into pDONR207 plasmid was used to generate point mutations in the three BED domains in order to determine whether BED domains were responsible of DNA binding. BED point mutations were done by substituting the two cysteines of the zinc finger to glycines in each of the three domains present in ZBED (**Supplementary Figure 2**). To this end, we used QuickChange lightning site-directed mutagenesis kit (Agilent Technologies) following the manufacturer’s instructions. Primers used are provided in **Supplemental Table 1**. Briefly, PCR conditions were initial denaturation: 95°C 2 min followed by 20 cycles of 95°C 15 s denaturation, 60°C 10 s annealing, and 68°C 4 min (30 s per kb) of extension step, with a final 68°C for 5 min. *E. coli* Top10 competent cells (Invitrogen™) were transformed with these plasmids and plated in LB agar with gentamicin selection and sent for sequencing to verify point mutation. Once the point mutation on each BED domain was confirmed, we proceeded to clone these constructs into a gateway plasmid 35S-eGFP-GWY (pB7FWG2 destination vector, https://gateway.psb.ugent.be) in order to transform *Agrobacterium tumefaciens* GV3101 cells for transient expression assays in *Nicotiana benthamiana*.

### Plant Growth

Three-week-old rice plants were used for drought stress assays and inoculation with *Magnaporthe oryzae* Guy11 strain. Temperature was maintained between 29°C day and 21°C night with a 16 h light cycle. Plants were grown in Neuhaus S soil mixed with poudzolane (2 L/70 L). Standard fertilization solution containing nitrogen source (75% NO−3/25% NH+4; 40 mg/L) was supplied every Monday, and plants were inoculated one or two days after fertilization.

Four-week-old *N. benthamiana* plants were used for transient expression of ZBED using *A. tumefaciens*. Plants were grown in a growth chamber under 16 h light cycle and 22/20°C day/night temperatures. Plants were kept under these conditions during the course of the experiments.

### Transient Expression Assays in *Nicotiana benthamiana*

Transient expression of recombinant proteins in *N. benthamiana* was performed as described previously with some modifications (Bos et al., 2006). Briefly, *A. tumefaciens* strains GV3101 were grown in LB medium at 28°C for 16 h and then centrifuged and resuspended in induction medium (10 mM MES (pH 5.6), 10 mM MgCl_2_, 150 mM acetosyringone) at an optical density at 600 nm (OD_600_) of 0.5 and incubated for 4 h at room temperature. Four-week-old *N. benthamiana* leaves (two per plant and five plants per biological rep, for a total of four biological reps) were infiltrated with *Agrobacterium* culture using a 1 ml needle-less syringe and evaluated for cell death phenotype at four and five days after infiltration (dai). For ZBED localization, samples were taken at two and three dai and observed under the confocal microscope.

### *Xanthomonas oryzae* Inoculation and Scoring of Disease Symptoms

Four bacterial strains of *X. oryzae* pv *oryzae* (*Xoo*) PXO99A, MaiI, BAI3 and X11-5A(ptal6-TalC) and one strain of *X. oryzae* pv *oryzicola* (*Xoc*) BLS256, were kindly provided by Boris Szurek (IRD, Montpellier) for this study. All bacterial strains were grown in PSA medium (10 g of peptone, 10 g of sucrose, 1 g of glutamic acid, 16 g of agar per liter of H_2_O) for three days at 28°C. Bacterial cultures were suspended in sterile distilled H_2_O at an OD_600_ of 0.2 for *Xoo* leaf-clip inoculations (Kauffman et al., 1973) and 0.5 for infiltrations using a needle-less syringe for *Xoc* (Reimers and Leach, 1991). Three to four-week-old N-1 rice leaves from four plants per biological rep, with a total of four biological reps, were inoculated, and plants were kept in a phytotron (SANYO) under 12 h light cycle and 28/21°C day/night temperatures. Symptoms were evaluated two weeks after leaf-clip inoculation for *Xoo* by measuring lesion length and one week after *Xoc* infiltration by evaluating both water soaking phenotype and lesion length.

### *M. oryzae* Inoculation and Scoring of Disease Symptoms

*M. oryzae* isolate Guy11 was cultured in rice agar for ten days (Berruyer et al., 2003). Spores were harvested in distilled water and filtered through two layers of nylon mesh. The spore concentration was adjusted to 30.000 spores/ml using a haemocytometer in 0.5% gelatin. Control plants were mock-inoculated with 0.5% gelatin. Inoculated and mock-inoculated plants (five per treatment) were transferred to an inoculation chamber at 25°C with 100% relative humidity for 16 h. Then plants were relocated in a phytotron 29°C day/21°C night in a 16 h light cycle. Disease symptoms were rated seven days after inoculation by measuring the percentage of susceptibility lesions, which are the number of susceptible lesions over the total number of lesions (susceptible plus resistant) multiplied by 100. The experiment was repeated four times.

### DAB Staining to Detect Hydrogen Peroxide *In Vivo* in *N. benthamiana*

Four leaves of four-week-old *N. benthamiana* plants were agroinfiltrated with GFP-ZBED, GFP-ZBED mutants (BED point mutations) and control vector carrying GFP only. Leaves were cut 48 hai, and the petiole was dipped into diaminobenzidine (Sigma D-8001) 1 mg/ml of water and kept overnight in dark conditions at room temperature. Next day, tissue was cleared with ethanol/chloroform (4:1) overnight at room temperature. Once the tissue was cleared, the leaves were placed in 60% glycerol, and browning of cells was observed under the light microscope and quantified using automated phenotyping. Briefly, images are represented in multidimensional arrays containing pixel intensity values using EBImage (Pau et al., 2010), and with these results we then performed a T-test and considered significant differences for p-values < 0.05. The experiment was repeated three times for a total of four biological replications.

### Preparation of Leaf Tissues for FRET-FLIM Analyses

*A. tumefaciens*-infiltrated *N. benthamiana* leaf disc samples (8-mm diameter, harvested 48 h post-infiltration) were vacuum-infiltrated in fixation solution (4% w/v) paraformaldehyde and 0.05 M (CH_3_)2AsO_2_Na and incubated for 20 min at 4°C. Samples were rinsed in TBS buffer (25 mM Tris-HCl (pH 7.5), 140 mM NaCl, 3 mM KCl) for 5 min. Permeabilization of fixed samples was performed by incubating in proteinase K buffer (50 mM Tris-HCl (pH 7.5), 100 mM NaCl, 1m MEDTA, 0.5% SDS, 200 mg/ml of proteinase K, Invitrogen) for 10 min at 37°C. Samples were rinsed in TBS buffer for 5 min. Nucleic acid staining was performed by vacuum infiltrating a 5 mM Sytox Orange (Invitrogen) solution (TBS buffer) and incubating samples for 30 min at room temperature in the dark. Fixed leaf discs were washed with and mounted on TBS buffer before observations on an inverted microscope (Eclipse TE2000E, Nikon). FRET-FLIM measurements were performed in at least 27 nuclei per construct (**Table 1**) as previously described (Le Roux et al., 2015).

**Table 1:** FRET-FLIM measurements showing that ZBED DNA-binding ability involves its BED domains.

Donor	Acceptor	τ^a^	SE	Δt^b^	n^c^	FRET^d^	p^e^
GFP-ZBED	−sytox	2.039	0.014	243	36	12	5.10–15
GFP-ZBED	+sytox	0.087	0.018		55		
GFP-ZBEDm1	−sytox	2.011	0.035	−1.766	30	0	0.72564379
GFP-ZBEDm1	+sytox	2.029	0.036		30		
GFP-ZBEDm2	−sytox	2.044	0.044	3.33	30	1.62	0.5891304
GFP-ZBEDm2	+sytox	2.011	0.043		30		
GFP-ZBEDm3	−sytox	1.972	0.036	−0.668	27	0	0.90472711
GFP-ZBEDm3	+sytox	1.978	0.043		28		
GFP-ZBEDm123	−sytox	2.135	0.029	3.204	41	1.5	0.40998124
GFP-ZBEDm123	+sytox	2.103	0.026		43		

a Mean lifetime (in nanoseconds). For each nucleus, average fluorescence decay profiles were plotted and fitted with exponential functions using a nonlinear square estimation procedure. Mean lifetime was calculated according to τ = Σ α_i_τ_i_²/ Σ α_i_τi with I(t) = Σ α_i_ e^−t/^τ^i^.

b Δt = τ_D_ − τ_DA_ (in nanoseconds).

c Total number of measured nuclei.

d Percentage of FRET efficiency: E = 1 − (τ_DA_/τ_D_).

e P value of the difference between the donor lifetimes in the presence and absence of acceptor (Student’s t test).

### RNA Extraction and RT-qPCR Analysis

RNA extraction was performed as described (Delteil et al., 2012). Quantitative RT-PCR was performed using LC 480 SYBR Green I Master Mix (Roche, Basel, Switzerland) and a LightCycler 480 instrument (Roche). The amount of rice RNA in each sample was normalized using actin (LOC_Os03g50890) as internal control. For *N. benthamiana* RNA in each sample was normalized using the housekeeping *NbPP2A* gene (Segonzac et al., 2011) as internal control. For each treatment we had four technical reps (each technical rep was the bulk of three leaf discs from three different plants); a total of four biological reps were done. Details of PCR primers are provided in **Supplemental Table 1**.

### Drought-Stress Protocols

Greenhouse drought stress assay was done as previously described with small modifications. Rice genotypes were sown on day 1 in 60 g of soil per pot (for soil composition and greenhouse conditions, see above). Each replicate consisted of 10 plants grown in 7.5 cm × 7.5 cm × 6.5 cm (h × w × l) pots, and a total of five replicates per treatment per genotype. Plants were watered daily and fertilized on day 5, starting from the second week after sowing. At day 23 (10:00), drought stress was imposed by withholding water. Three days later (day 26; 10:00), drought-stressed plants were rehydrated prior to inoculation, and no fertilization was applied. The experiment was repeated twice for a total of three biological reps. This protocol was used to apply drought on greenhouse-grown plants shown in **Figure 4B**. Measurements in control plants indicated that this treatment significantly (T-test p<0,01) reduced Relative Water Content by ~15%, and published data indicate that drought-responsive genes were induced upon this treatment (Bidzinski et al., 2016).

For drought field experiments, a confined rain-out shelter field facility at CIAT (International Center for Tropical Agriculture, Cali Colombia) was used as previously described (Khong et al., 2015). Briefly, field trials were laid as a randomly complete block design with three replicates. Drought stress was imposed at panicle initiation (56 days after direct seeding) and continued until severe leaf rolling and wilting started in non-transgenic controls (three weeks approximately). Plants were re-watered until physiological maturity. Leaf rolling scores, plant height (centimeters), single-plant dry biomass (grams), and single-plant yield were recorded following the Standard Evaluation System for Rice (Gregorio et al., 1997). This protocol was used for drought measurements in field experiments shown in **Figure 4B**.

### Salinity Stress Assay

Rice seeds were dehulled and surface-sterilized in 70% ethanol for 3 min. Ethanol was discarded, and seeds were submerged in 40% bleach for 30 min with shaking (discard bleach), then seeds were rinsed five times with sterile H_2_O under the hood. Ten surface-sterilized seeds per genotype were sown in one autoclaved magenta box containing 100 ml MS (Murashige & Skoog; Duchefa Biochemie) medium 4.3 g/l, with 8 g/l of agar amended with 120 mM NaCl (Electric conductivity 12dSm^−1^) for the salinity stress treatments or without NaCl for the controls (IRRI, 2014). Each genotype was sown under control MS and MS + NaCl in six independent experiments with similar results. The salt tolerant Pokkali accession which has the major QTL for salt tolerance *Saltol* was used as control (Thomson et al., 2010). Seed germination rates together with shoot and root length were measured three weeks after sowing. Root and shoot biomass were determined for three-week-old plants after drying in an oven at 65°C for one week.

## Results

### Identification of ZBED Protein Interactors

**Figure 1:**
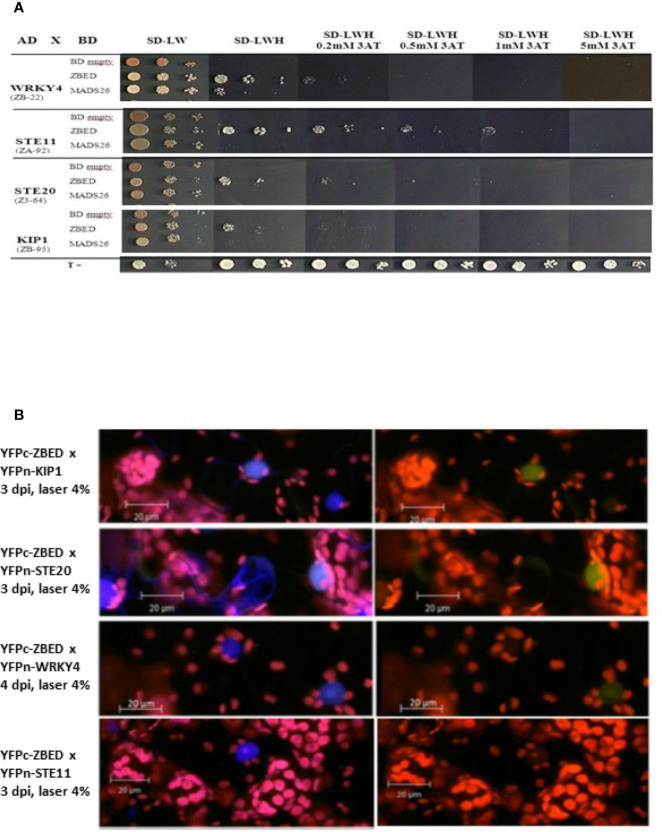
Identification of ZBED-interacting proteins. **(A)** Screening of two rice yeast two-hybrid cDNA libraries (CO39 and RCL1) with ZBED led to the identification of several putative interacting partners. WRKY4, STE11, STE20, and KIP1 were found to interact with ZBED in yeast cells. Yeasts co-expressing BD-ZBED with either AD-WRKY4, AD-STE11, AD-STE20, or AD-KIP1 were plated on selective media (SD-LWH) with different concentrations of 3AT. Empty pGBKT7-BD (BD-empty) was used as negative bait control. **(B)** Bimolecular Fluorescent Complementation (BiFC) assay was used to confirm yeast two-hybrid interactions (right panels). Transient co-expression of YFPc-ZBED either with YFPn-KIP1, YFPn-STE20, and YFPn-WRKY4, led to the detection of a nuclear fluorescent signal. Lack of YFP fluorescence upon co-expression of YFPc-ZBED with YFPn-STE11 suggests that these two proteins do not associate *in planta*. DAPI staining was used for nuclear staining (left panels). Observations were performed on *N. benthamiana* leaf samples collected three and four days post infiltration (dpi).

In order to gain a better understanding of ZBED’s molecular functions and predict the biological processes in which it could be involved, we performed a yeast two-hybrid (Y2H) screening to look for ZBED-interacting proteins in rice. We screened two rice libraries (Cesari et al., 2013; see *Materials and Methods*), using ZBED as bait. Among the various prey clones isolated, we identified KIP1 (LOC_Os01g07370), a kinase interacting protein predominantly expressed in pollen (Skirpan et al., 2001), STE11 (LOC_ Os01g50400), STE20/MAPK3 (LOC_Os12g0225; Jouannic et al., 1999) and WRKY4 (LOC_ Os03g55164), a transcriptional activator of rice defense responses against *Rhizoctonia solani* (Wang et al., 2015) (**Figure 1A**; ZBED auto-active controls **Supplementary Figure 1**). To confirm *in planta* the Y2H interacting data, we used a Bimolecular Fluorescent Complementation (BiFC) assay. A nuclear yellow-fluorescent protein (YFP) signal was detected when YFPc-ZBED was co-expressed either with YFPn-KIP1, YFPn-STE20 or YFPn-WRKY4 although it seems weaker than with KIP1 and STE20, but not with YFPn-STE11, confirming three of the four interactions detectable in yeast cells (**Figure 1B**).

### ZBED Localizes in the Plant Nucleus and Binds DNA

**Figure 2:**
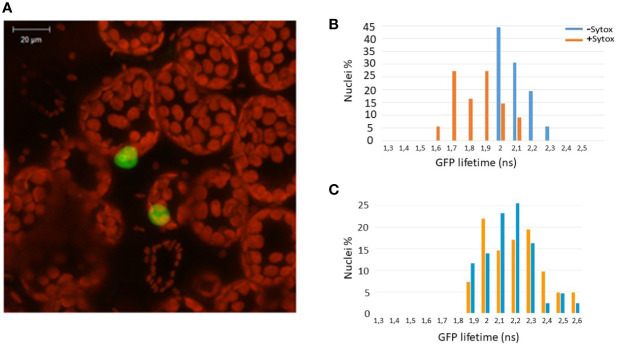
ZBED localizes to the nucleus and binds DNA. The GFP-ZBED fusion protein was transiently expressed in *N. benthamiana*. **(A)** GFP-ZBED accumulates in the plant nucleus; DNA binding using FRET/FLIM with or with Sytox (blue and orange bars respectively) with wild-type **(B)** and a mutated version of ZBED with point mutations of the cysteine to a glycine in each of three BED domains **(C)**.

Since Zf-BED domains are hypothesized to bind DNA (Aravind, 2000), and considering our BiFC experiments (**Figure 1B**) indicating that ZBED is detected in the nucleus, we investigated the subcellular localization of ZBED and its putative DNA-binding ability. For this, ZBED was N-terminally fused to green fluorescent protein (GFP-ZBED) and transiently expressed in *N. benthamiana*. Laser-scanning confocal microscope analysis led to the detection of GFP fluorescence exclusively in plant nuclei indicating that ZBED is specifically targeted to this subcellular compartment (**Figure 2A**). A FRET-FLIM (fluorescence resonance energy transfer-fluorescence lifetime imaging microscopy) assay (Le Roux et al., 2015) was performed to detect protein-nucleic acid interactions. GFP-ZBED was used as a donor fluorophore. A DNA-binding fluorescent dye (Sytox Orange) treatment performed on fixed plant material converts nuclear nucleic acids to FRET acceptors. In the absence of Sytox Orange treatment, an average GFP lifetime of 2.1 nanoseconds (ns) was measured (**Figure 2B**). A significant decrease of the GFP-ZBED lifetime was observed (1.8 ns), in Sytox orange-treated samples, indicating a close association of the GFP-ZBED partner (donor) and stained DNA (acceptor) due to FRET (**Figure 2B**). Because the cysteines and histidines are predicted to form a zinc-finger that binds DNA, each of the three BED domains were mutated separately by substituting their two conserved cysteines (which form zf) for two glycines to abolish DNA binding (**Supplementary Figure 2**). A single point mutation in any of the BED domains abolished the physical interaction between ZBED and DNA (**Table 1**), indicating that the integrity of each of ZBED’s zf-BED domains is strictly required for proper DNA-binding activity. The absence of DNA-binding in the zf-BED triple-mutant is depicted in **Figure 2C**. Together, our data indicate that ZBED localizes in the nucleus and binds DNA.

### ZBED Induces Cell Death and Activates Basal Defense Responses in *N. benthamiana*

**Figure 3:**
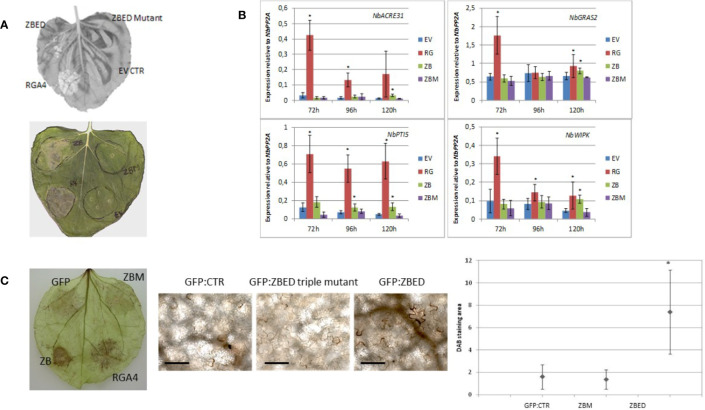
ZBED phenotypes related to basal immunity in *N. benthamiana*. **(A)** Transient expression of ZBED in *N. benthamiana* induces cell death. Transient expression of ZBED caused cell death four to five days after infiltration. ZBED mutants (ZBM) or GFP empty vector (EV) did not cause cell death. Positive control autoactive rice resistance gene *RGA4* (R4-RGA4). **(B)** ZBED induces PAMP Triggered Immunity (PTI) in *N. benthamiana* as measured by RT-qPCR with four different PTI markers: *NbACRE31*, *NbGRAS2*, *NbPTI5*, and *NbWIPK*. ZBED triple mutant (ZBM) behaved like the GFP empty vector (EV) negative control at all time points and with all markers. Positive control autoactive rice resistance gene *RGA4* (RG) (T-test *p < 0.05). **(C)** ZBED induced reactive oxygen species (ROS) measured with DAB staining, while ZBED mutant (ZBM) and GFP empty vector (EV) control did not. Quantification was done using EBImage (Pau et al., 2010; T-test *p < 0.05); scale bar is 100 µm.

While using *N. benthamiana* as a heterologous system to determine ZBED localization, we noticed that after 4–5 days, there was a cell death phenotype caused by ZBED transient overexpression (**Figure 3A**), although this phenotype was not as strong as with the rice resistance protein RGA4 used as a positive control. This weak cell death was abolished with all ZBED-mutants, even when a single BED domain was mutated (**Figure 3A** shows phenotype with ZBED-triple mutant), suggesting that their DNA-binding activities are important for the cell death phenotype. To characterize the mechanisms by which ZBED was inducing cell death in *N. benthamiana*, we first checked whether ZBED was able to induce basal immunity, using four PTI-related marker genes: *NbACRE31*, *NbGRAS2*, *NbPTI5*, and *NbWIPK* (Hao et al., 2014). We measured their expression by RT-qPCR at three different time points (72, 96, and 120 h) after ZBED expression in *N. benthamiana* leaves. As shown in **Figure 3B**, ZBED overexpression enhanced the expression level of all four marker genes suggesting that it activates PTI, although to lower levels than with the RGA4 positive control. However, PTI induction was no longer detected with the ZBED-mutants, suggesting that this induction depends on the ability of ZBED to bind DNA (**Figure 3B**). Additionally, we explored whether ZBED was able to activate the production of reactive oxygen species (ROS), a well described readout of basal immunity. For this, *N. benthamiana* leaf samples expressing either GFP-ZBED, GFP-ZBED-mutant, or GFP alone were collected 48 h after *Agrobacterium* infiltrations and subjected to a DAB staining procedure. Transient expression of GFP-ZBED induced ROS while both the GFP-ZBED mutant and GFP did not (**Figure 3C**).

### Role of ZBED in Resistance to the Bacterial Pathogen *Xanthomonas oryzae*

Individual T2 plants (homozygous overexpressor and non-overexpressor–azygous) from three independent lines carrying single copy insertions were selfed, and *ZBED* overexpression was confirmed in the T3 progeny (see *Material and Methods*; Grand et al., 2012). ZBED overexpressor lines (ZBED-OX1, ZBED-OX2, and ZBED-OX3) were more resistant to *M. oryzae* (Kroj et al., 2016) than the non-overexpressor azygous lines (ZBED 1/0, ZBED 2/0 and ZBED 3/0 respectively). Our initial hypothesis postulated that ZBED could be involved in *X. oryzae* resistance (Zuluaga et al., 2017) functioning as a decoy for pathogen effectors, because the rice gene Xa1 has an integrated BED domain (Yoshimura et al., 1998; Zuluaga et al., 2017). We used the strongest ZBED overexpressor (ZBED-OX1) and its respective azygous line (ZBED 1/0) (Kroj et al., 2016) to test whether ZBED conferred resistance against different strains of this bacterial pathogen. Four different strains of the vascular pathogen *X. oryzae* pv *oryzae* (*Xoo*) (PXO99A, MaiI, BAI3, X11-5A (ptal6-TalC)) and one strain of *X. oryzae* pv *oryzicola* (*Xoc*) BLS256 were used. However, there was no effect on pathogenicity after inoculation of the ZBED-OX1 plants, compared to ZBED 1/0 plants, suggesting that ZBED-overexpressing lines do not show resistance to these bacterial pathogens (**Supplementary Figure 3**).

### Rice ZBED Overexpressor Lines Are Resistant to Drought but Not to Salt Stress

**Figure 4:**
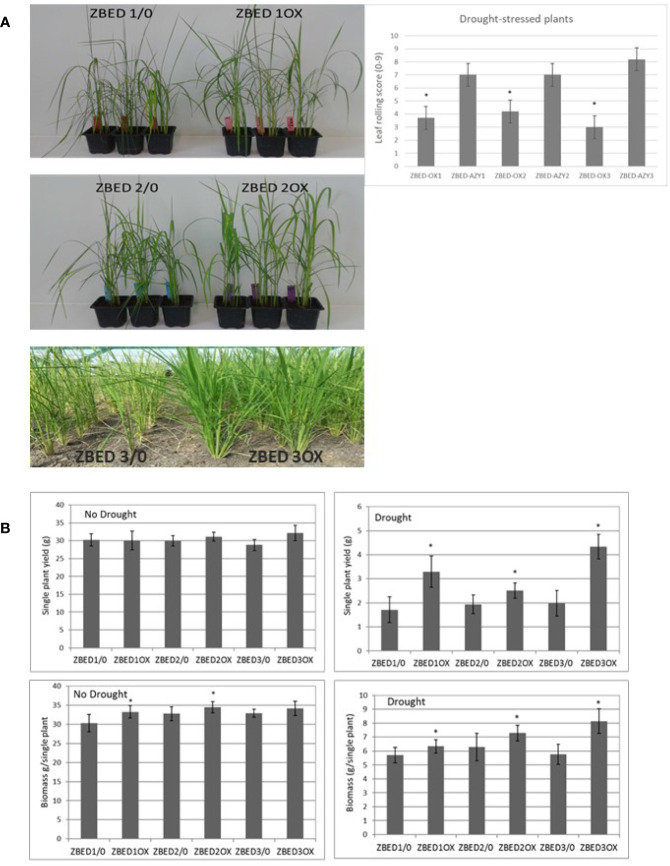
ZBED over-expression enhances drought tolerance. **(A)** Rice ZBED overexpressor lines are resistant to drought stress both in controlled (greenhouse) and non-controlled (Field, CIAT, Colombia) conditions; leaf-rolling score graph shows that ZBED-OX lines have less wilting symptoms than the azygous controls three days after water was withheld, using a scale from 0: non leaf rolling to 9: leaf completely wilted (n = 3; T-test *p < 0.05). **(B)** Agronomical traits in three independent ZBED overexpressor lines and their respective azygous lines under optimal water conditions and drought in the confined field rainout shelter facility (CIAT, Colombia). Single plant yield and single plant biomass are significantly higher in ZBED overexpressor lines under drought stress than in their respective azygous controls (T-test *p < 0.05).

Because of the frequent negative cross-talk between disease resistance and abiotic stress responses, we hypothesized that ZBED overexpressor (ZBED-OX) plants with enhanced resistance against *M. oryzae* (Kroj et al., 2016), might be less tolerant to abiotic stresses. Thus, we tested whether ZBED conferred susceptibility to drought and salinity stresses. Drought experiments were done in both the greenhouse and field (**Figure 4A**). Three independent transformation lines (ZBED-OX1, ZBED-OX2, and ZBED-OX3, collectively called ZBED-OX), together with their respective azygous lines (ZBED 1/0, ZBED 2/0, ZBED 3/0, collectively called AZY), were tested under controlled field conditions using a confined rain-out shelter field facility at CIAT (Centro Internacional de Agricultura Tropical, Colombia). ZBED-OX lines were significantly more resistant to drought-induced stress than the corresponding AZY lines as measured by different agronomical traits (**Figure 4B**). Single plant yield measured as grams of seeds produced per plant, was significantly higher in ZBED-OX compared to AZY lines under drought stress, although it must be noticed that drought-stressed plants had ten times less yield than well-watered plants, regardless of the genotype (**Figure 4B**). Under control conditions, single plant biomass measured as foliage dry weight, was higher for ZBED-1OX and 2OX compared to the azygous controls, except for 3OX plants. Drought had a more negative impact on AZY lines biomass thane on that of ZBED-OX lines, especially ZBED-3OX. As a result, biomass was significantly higher in all ZBED-OX lines compared to all AZY lines (**Figure 4B**) even though all genotypes (OX and AZY) showed an important decline (around ten times), in plant biomass after drought stress. Moreover, drought stress did not seem to have an impact on panicle length or number of productive tillers (t-test > 0.05; **Supplementary Figure 4**). To gain a better understanding on how ZBED was contributing to drought resistance, we further challenged ZBED-OX plants using a well-established drought protocol under controlled greenhouse conditions (Bidzinski et al., 2016). Greenhouse experiments showed that leaf rolling and wilting during the drought-stress peak were lower in ZBED-OX lines than in their corresponding AZY lines (**Figure 4A**). Because increased resistance to drought is often associated with salinity resistance due to the overlap of plant responses mechanisms to both stresses (Roychoudhury et al., 2013), we tested whether ZBED-OX lines conferred enhanced tolerance to salinity. To this end, we performed *in-vitro* assays by sowing seeds on MS medium supplemented with 120mM NaCl (corresponding to an electrical conductivity of dSm^−1^ 12.2) which has been reported to cause a decrease in rice germination and seedling survival (Senguttuvel et al., 2013). Our data indicate that ZBED overexpression did not increase tolerance to salinity stress at the seedling stage (**Supplementary Figure 5**). Thus, plants overexpressing ZBED showed elevated resistance against *M. oryzae*, enhanced tolerance to a mild drought episode and did not develop any phenotype in response to salt at the seedling stage.

### ZBED Positively Regulates Stress-Responsive Genes

**Figure 5:**
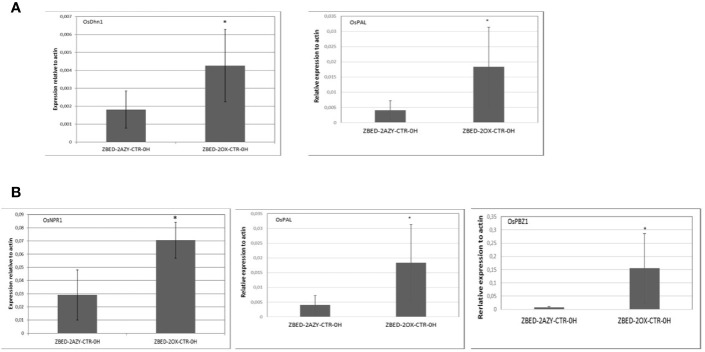
Constitutive expression of drought marker genes and disease-related marker genes in ZBED over-expressor. **(A)** Quantification of abiotic stress and **(B)** disease-related markers in one overexpressor line and its respective azygous, under control conditions (no inoculation/no drought). ZBED overexpressor (ZBED-OX) line showed a higher expression of *OsDhn1*, *OsMAPK5*, *OsNPR1*, *OsPAL*, and *OsPBZ1* (T-test *p < 0.05; bars represent SD for four biological reps) than its azygous control (AZY).

To gain a better understanding of the mechanism by which ZBED was conferring drought tolerance and disease resistance, we measured constitutive expression of stress-related markers under normal growth conditions (no inoculation and no drought stress). To this end, we performed RT-qPCR of six key regulatory genes of abiotic stress: *OsDhn1*, *Oshox22*, *OsDREB2A*, *OsMAPK5*, *OsMYB4* and *OsNAC6* (Xiong and Yang, 2003; Nakashima et al., 2007; Agarwal and Jha, 2010; Cui et al., 2011; Zhang et al., 2012; Kumar et al., 2014). We found that contrary to the AZY lines, ZBED-OX lines had significantly higher levels of dehydrin *OsDhn1* and *OsMAPK5* expression under control conditions (t-test p < 0.05; **Figure 5A**). Next, we quantified the expression level of five central regulators and marker genes of disease resistance: *OsNPR1*, *OsPAL*, *OsPBZ1*, *OsPR1*, and *OsWRKY45* (Jwa et al., 2001; Yuan et al., 2007; Mitsuhara et al., 2008; Qiu and Yu, 2009; Tonnessen et al., 2014). We observed differential expression of components of the salicylic acid (SA) pathway; both the master regulator *OsNPR1* (Yuan et al., 2007) and the *OsPBZ1* gene had significantly higher expression in overexpressor plants compared to azygous controls (t-test p < 0.05; **Figure 5B**). Additionally, the *OsPAL* gene a key component of the phenylpropanoid pathway was strongly expressed in overexpressor plants compared to azygous controls. Thus, ZBED-OX plants showed elevated expression of both abiotic and biotic key regulatory genes expressed under control conditions, indicating that ZBED positively regulates some stress-responsive genes which might prime plants against both episodes of mild drought and *M. oryzae*.

### Characterization of Resistance to *M. oryzae* After Drought Stress

**Figure 6:**
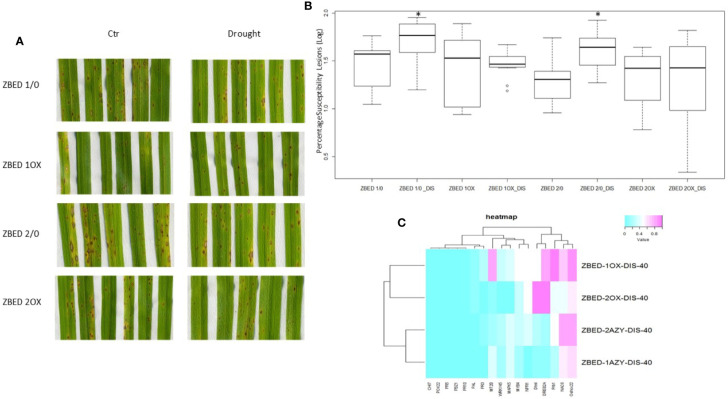
Disease resistance in ZBED-OX after drought stress. **(A)** Phenotypic evaluation of ZBED plants inoculated with *M. oryzae* after drought stress. ZBED-OX lines have smaller and less susceptible lesions after drought stress than the AZY lines. **(B)** ZBED overexpressor lines subjected to drought stress are significantly more resistant to *M. oryzae* than the azygous controls (ANOVA, Tukey HSD *p < 0.05). **(C)** Heatmap of marker genes for both abiotic and biotic stresses occurring simultaneously at 40 h after inoculation (hai). Except for *OsNPR1*, all the other biotic stress marker genes tested were not differentially expressed in ZBED overexpressor lines 1OX and 2OX compared to their respective azygous lines 1/0 and 2/0 under both drought and *M. oryzae* infection. However, the abiotic stress markers *DREB2A* and *Dhn1* show a higher expression in ZBED-OX plants compared to AZY.

We and others have demonstrated that rice plants are more susceptible to *M. oryzae* after a drought-stress episode, a phenomenon called drought-induced susceptibility (DIS) (Bonman, 1992; Bidzinski et al., 2016). Because ZBED-OX lines showed increased resistance against *M. oryzae* and tolerance to mild drought stresses occurring separately, we wanted to test whether ZBED-OX lines were able to confer resistance against the rice blast fungus after a mild drought-stress episode. For this, 22-day-old plants were submitted to a mild drought stress for three days. On the third day, plants were re-watered 4 h prior to inoculation at which point they no longer showed a drought-stress phenotype (leaf rolling or wilting). Plants were inoculated with *M. oryzae* and symptoms were scored seven days after inoculation. We observed that ZBED-OX genotypes remained significantly more resistant to *M. oryzae* than the corresponding AZY lines (ANOVA, Tukey HSD p < 0.05) despite the drought stress prior to inoculation, suggesting that mild drought did not interfere with ZBED-triggered resistance (**Figures 6A, B**).

Leaf tissue was collected at 40, 64, and 88 h after inoculation (hai), and RT-qPCR analysis for both abiotic and biotic marker genes were done in order to test if the differential expression of these genes could explain pathogen resistance under drought in ZBED-OX plants. We observed that both ZBED-OX lines tested showed higher expression than azygous plants of the stress marker genes *OsDhn1*, *OsDREB2A* and *OsNPR1* after a drought stress episode at early stages of the interaction (40 hai; **Figure 6C**). Additionally, there was a change in gene expression throughout infection, with the drought marker genes *OsMAPK5*, *OsMYB4*, and *OsNAC6* expressed higher in OX lines compared to AZY lines at 64 and 88 hai (**Supplementary Figures 6A–C**). Conversely, the biotic stress marker genes that we studied were not differentially expressed between ZBED-OX and AZY lines throughout the interaction, suggesting that they do not contribute to the observed resistance phenotype (**Supplementary Figures 6D–F**).

## Discussion

### ZBED Molecular Function

The molecular function of BED domain remains elusive in plants. Only one report indicates that they bind DNA (Coupe and Deikman, 1997), and they were recently shown to direct proteins into the plasma membrane (Khong et al., 2015). Here we demonstrate that ZBED localizes to the nucleus and binds DNA in a heterologous system. ZBED DNA-binding activity seems to require the integrity of each of the three cysteine residues in the zinc-fingers of the BED domains of ZBED (**Table 1**). This observation is consistent with previously published data showing that BED domains bind DNA in Arabidopsis (Bundock and Hooykaas, 2005). Whether the ability of ZBED to bind DNA requires zinc ions (Zn^2+^), like for other Zn-Finger proteins, remains to be determined. In animals, BED proteins display various molecular functions, including transcriptional activation (Wang et al., 2013). In support of such function in rice, transient expression of wild-type ZBED in *N. benthamiana* induced the expression of several stress-related genes, whereas the triple-mutated ZBED did not (**Figure 5**). Transcriptional regulation activity of ZBED is consistent with the observation that ZBED interacts in the nucleus with WRKY4 (**Figure 2**) previously described as a transcriptional activator (Wang et al., 2015).

In animals, only a few reports identified protein partners of BED proteins (Chen et al., 2009). To date in plants, no protein partner has been described for a BED-containing protein. Using Y2H and BiFC assays, we found that ZBED interacts in the nucleus with the MAP kinase STE20 (Jouannic et al., 1999), the transcriptional regulator WRKY4 (Wang et al., 2015) and the kinase interacting protein KIP1. Interestingly, two thirds of all MAPK localize to both the cytosol and the nucleus where most of their substrates identified so far are transcription factors (Bigeard and Hirt, 2018). For instance, in rice the OsBWMK1 localizes predominantly to the cytosol, but is translocated into the nucleus within 1 to 12 h after treatment with H_2_O_2_ and SA (Koo et al., 2007). This ZBED-interacting protein network shades some light into its possible molecular function. One can speculate that putative ZBED transcriptional activity could be modulated by kinase activities and coordinated through interactions with additional partners including transcription factors like WRKY4.

Additionally, we determined that ZBED, when transiently expressed in *N. benthamiana*, induces components of basal defense such as PTI, ROS and cell death. Remarkably, ZBED overexpression in rice plants did not trigger cell death. Different explanations can account for this result. On the one hand, it suggests that rice, not *N. benthamiana*, has a negative regulator suppressing the cell death-inducing activities of ZBED. This regulator could be similar to the product of the rice disease resistance gene RGA4, used here as a positive control, that causes severe cell death when transiently expressed in *N. benthamiana* but induces no phenotype when overexpressed in rice (Cesari et al., 2014a). On the other hand, it might be an artefact of expressing ZBED in a heterologous system, where high amounts of protein could cause the cell death phenotype.

### Role of ZBED in Resistance to *Xanthomonas oryzae*

Recently, it has been demonstrated that decoy proteins with a role in effector recognition can be integrated into plant immune receptors, giving rise to the integrated decoy model (Cesari et al., 2014b). In rice, a BED domain was found integrated with the *Xa1* resistance protein, which confers resistance against *Xanthomonas oryzae* (Yoshimura et al., 1998). Thus, it was tempting to hypothesize that the BED domain of Xa1 acts as a decoy whose targeting by *X. oryzae* effectors would trigger resistance response activation against this pathogen in rice (Zuluaga et al., 2017). According to this model, ZBED with its BED domains could represent a virulence target for *X. oryzae* effectors. However, we did not observe any difference in pathogenicity after inoculation with *Xoc* or *Xoo* isolates between ZBED-OX and AZY lines (**Supplementary Figure 3**). Our results suggest that ZBED-OX lines do not show resistance to these bacterial pathogens, invalidating the model we recently proposed (Zuluaga et al., 2017). Consistent with this observation, three resistance genes necessary to confer resistance against a wide range of the yellow rust fungus, which is unrelated to *Xanthomonas*, have recently been shown to carry a BED domain in their N-terminus (Marchal et al., 2018). Thus, the BED domain found in resistance proteins may not act as a decoy but could rather function as a downstream signaling or localization module required for some resistance proteins to activate immune responses.

### Rice ZBED-OX Lines Are Resistant to Rice Blast Even After Drought Stress

ZBED-OX plants showed increased drought tolerance after a mild drought as well as disease resistance. Thus, the *ZBED* gene represents one of the few cases where disease resistance and drought tolerance are simultaneously improved, similar to *OsMADS26*, *MAPKK10.2*, *OsNAC6*, *OsCPK4*, *OsCPK10*, *WRKY11*, or *OsHAP2E* (Nakashima et al., 2007; Campo et al., 2014; Alam et al., 2015; Khong et al., 2015; Bundó and Coca, 2016; Bundó and Coca, 2017; Ma et al., 2017; Lee et al., 2018). The mechanisms by which ZBED-OX increases disease resistance and drought tolerance were explored in this study. Several disease-related marker genes were highly induced in ZBED-OX plants under normal growth and in the absence of infection (**Figure 5**). For instance, expression of *OsNPR1* encoding for a key regulator of SA-mediated resistance in rice (Yuan et al., 2007) was significantly increased in ZBED-OX. Likewise, *OsPAL* that encodes for a key enzyme in the SA biosynthesis and the phenylpropanoid pathway (Tonnessen et al., 2014) was significantly induced. Additionally, two executors of defense, *OsPBZ1* and *PRb1*, were induced as well (**Figure 5**). Although gene expression was not measured in the field, it is noteworthy that yield was not impacted in ZBED-OX plants (**Figure 4B**) despite a possible constitutive overexpression of these stress-related genes.

After fungal infection, the expression of disease-related marker genes was not strongly different between ZBED-OX and AZY. This suggests that ZBED is modifying plant’s immunity before and not after infection, leading to a priming effect which could be sufficient to increase resistance (Vergne et al., 2010). Another possibility is that other disease-resistant pathways are induced after pathogen infection, but that we did not use the correct markers to detect them. With respect to drought-related markers measured, we observed that *OsDhn1* and *OsMAPK5* were constitutively expressed in well-watered, non-inoculated ZBED-OX plants (**Figure 5**). The *OsDhn1* gene has been shown to protect rice plants against drought and salinity stress by scavenging ROS (Kumar et al., 2014). Quite surprisingly, the *OsMAPK5* which overexpression induces salt, drought, and cold resistance but negatively modulates disease resistance against *M. oryzae* (Xiong and Yang, 2003), was also constitutively expressed in ZBED-OX lines, without an evident trade-off in disease resistance against rice blast. Thus, more gene expression studies will be required to fully understand how ZBED overexpression enhances both resistance to the fungal pathogen (but not bacterial) and tolerance to drought (but not salt or osmotic stress).

One last important result of our work is that we show that the disease resistance conferred by ZBED is still functional after drought stress, demonstrating that it is possible to build such robust resistance. This was unexpected because it was previously shown that drought stress slightly reduces the constitutive expression of defense genes and strongly diminishes its induction under infection (Bidzinski et al., 2016). It is important to highlight that this is one of the few studies where the outcome of two stresses applied sequentially was analyzed. Indeed, most of the effects of one given transgene on resistance/tolerance to different stresses have been done by applying and evaluating the outcome of each stress separately. Evaluating rice plants overexpressing *OsMADS26*, *OsNAC6*, *OsCPK4*, *OsCPK10* among others, under both drought and pathogen infection occurring simultaneously or sequentially, will indicate whether ZBED behaviour is unique of its kind.

## Conclusions

Using different approaches, we broaden the knowledge on the molecular function of BED domains in plants. Since these domains are now frequently found in plant resistance genes, our work will be of interest to groups working on the molecular function of resistance genes. Additionally, we demonstrate that overexpression of ZBED gene in rice, represents one of the few cases where disease resistance to the rice blast fungus is maintained even after a drought episode. This provides the first example to our knowledge of transgenic plants that display robust disease resistance under abiotic stress occurring simultaneously.

## Data Availability Statement

The raw data supporting the conclusions of this article will be made available by the authors, without undue reservation, to any qualified researcher.

## Author Contributions

AZ performed experiments, analyzed data, and wrote the paper. PB, AD, EC, BC, MG, MI, LD, and CM performed experiments. TK, BS, and RK provided conceptual advice. J-BM conceived the project, analyzed data, and wrote the paper. All authors contributed to the article and approved the submitted version.

## Funding

This publication has been written with the support of the AgreenSkills+ fellowship programme which has received funding from the EU’s Seventh Framework Programme under grant agreement N° FP7-609398 (AgreenSkills+) for PZ. This work was supported by an INRA project from the SPE division (Decoy) as well as the Agropolis and Cariplo Foundations under the reference « Rice Connections » 1201- 001 which is supported by the French ANR program “Investissement d’Avenir” ANR-10-LABX-0001-01.

## Conflict of Interest

The authors declare that the research was conducted in the absence of any commercial or financial relationships that could be construed as a potential conflict of interest.

## Supplementary Material

Supplementary Figure 1 Yeast two-hybrid ZBED auto-activation tests. Yeasts co-expressing BD-ZBED with AD-empty were plated on selective media (SD-LWH) with different concentrations of 3AT to test for auto-activation. A SAP9 protein from another project was added as an auto-activation positive control.

Supplementary Figure 2 Point mutations in BED domains. Two point-mutations were done in each of the BED domain to substitute the cysteine for glycine, to determine whether the BED domains where responsible for DNA-binding.

Supplementary Figure 3 Determining if ZBED is a target for *Xanthomonas oryzae* effectors. ZBED azygous (ZBED 1/0) and ZBED overexpressor (ZBED 1OX) inoculated with *Xanthomonas oryzae pv oryzae* (Xoo) PXO99A **(A)**, MaiI **(B)** and *X. oryzae oryzicola* (Xoc) BLS256 **(D)**. There is no significant difference (T test p > 0.05) in pathogenicity between ZBED overexpressor lines *vs* azygous controls with any of the Xoo or Xoc tested.

Supplementary Figure 4 Evaluation of agronomical traits in rice ZBED overexpressor *vs* ZBED azygous lines in the field (CIAT, Colombia). Number of productive tillers and panicle length are not significantly different ZBED overexpressor lines under drought stress than in their respective azygous controls (T test p > 0.05).

Supplementary Figure 5 ZBEDOX and AZY seeds were germinated in MS media, supplemented with 120 mM NaCl to induce salt stress. Pokkali seedlings were used as the resistant control. Relative weight of salt stressed plants over control plants (MS media no-salt added) is shown for root biomass (left) and shoot biomass (right). There is no significant difference between overexpressors or azygous ZBED lines in salt resistance at seedling stage (T test p > 0.05).

Supplementary Figure 6 Heatmap of marker genes for abiotic stress at 40 **(A)**, 64 **(B)** and 88 **(C)** h after inoculation (hai) under drought stress (DIS) or no drought (CTR). At 40 hai, abiotic stress markers DREB2A and DhnI show a higher expression in OX plants compared to AZY, while at 64 hai MYB4 and MAPK5 are more expressed in ZBED-OX than ZBED-AZY independently of whether they are inoculated or not. Finally, at 88 hai the expression of MAPK5, NPR1, NAC6, and MYB4 is higher in ZBED-OX plants in both drought and control plants. Heatmap of marker genes for biotic stress at 40, 64, and 88 h after inoculation (hai) **(D, E**, and **F** respectively) under drought stress (DIS) or no drought (CTR). Biotic stress markers tested are not differentially expressed in ZBED overexpressor lines 1OX and 2OX compared to their respective azygous lines 1/0 and 2/0 under both drought and *M. oryzae* infection.

## Abbreviations

PAMPs, pathogen-associated molecular patterns; PTI, PAMP-triggered immunity; ROS, reactive oxygen species; SA, salicylic acid; PR, pathogenesis-related genes; ABA, abscisic acid; JA, jasmonic acid; MAPK, mitogen-activated protein kinase; *Xoo, Xanthomonas oryzae;* OsCPK, calcium-dependent protein kinases; Y2H, yeast two-hybrid (Y2H); KIP, kinase interacting protein; BiFC, Bimolecular Fluorescent Complementation; YFP, yellow-fluorescent protein; GFP, green-fluorescent protein; FRET-FLIM, fluorescence resonance energy transfer-fluorescence lifetime imaging microscopy; OX, Overexpressor; AZY, Azygous; DAI, days after infiltration; ZF, zinc-finger.
