# Supplementary material for: The Rice DNA-Binding Protein ZBED Controls Stress Regulators and Maintains Disease Resistance After a Mild Drought
Source: Front Plant Sci. 2020 Aug 18;11:1265. doi: 10.3389/fpls.2020.01265 (PMC7461821; doi:10.3389/fpls.2020.01265)
Supplement: Supplementary Figure 1 — Yeast two-hybrid ZBED auto-activation tests. Yeasts co-expressing BD-ZBED with AD-empty were plated on selective media (SD-LWH) with different concentrations of 3AT to test for auto-activation. A SAP9 protein from another project was added as an auto-activation positive control. [file Presentation_1.pptx]

## Slide 1
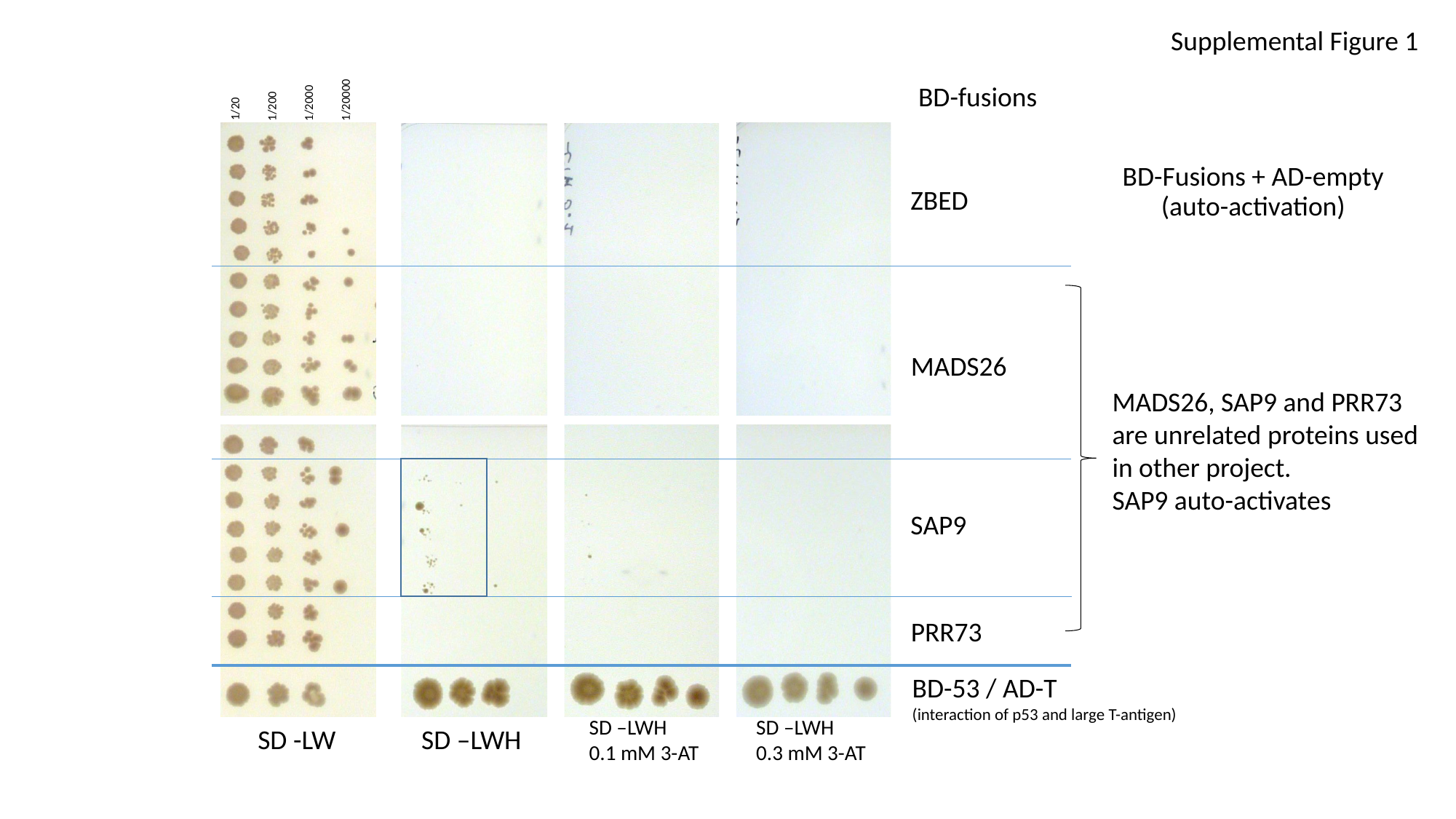

Supplemental Figure 1
BD-fusions
1/20000
1/2000
1/200
1/20
# BD-Fusions + AD-empty (auto-activation)
ZBED
MADS26
MADS26, SAP9 and PRR73
are unrelated proteins used
in other project.
SAP9 auto-activates
SAP9
PRR73
BD-53 / AD-T
(interaction of p53 and large T-antigen)
SD –LWH
0.1 mM 3-AT
SD –LWH
0.3 mM 3-AT
SD -LW
SD –LWH
