# Supplementary figures and images for: The Rice DNA-Binding Protein ZBED Controls Stress Regulators and Maintains Disease Resistance After a Mild Drought
Source: Front Plant Sci. 2020 Aug 18;11:1265. doi: 10.3389/fpls.2020.01265 (PMC7461821; doi:10.3389/fpls.2020.01265)

## Slide 1
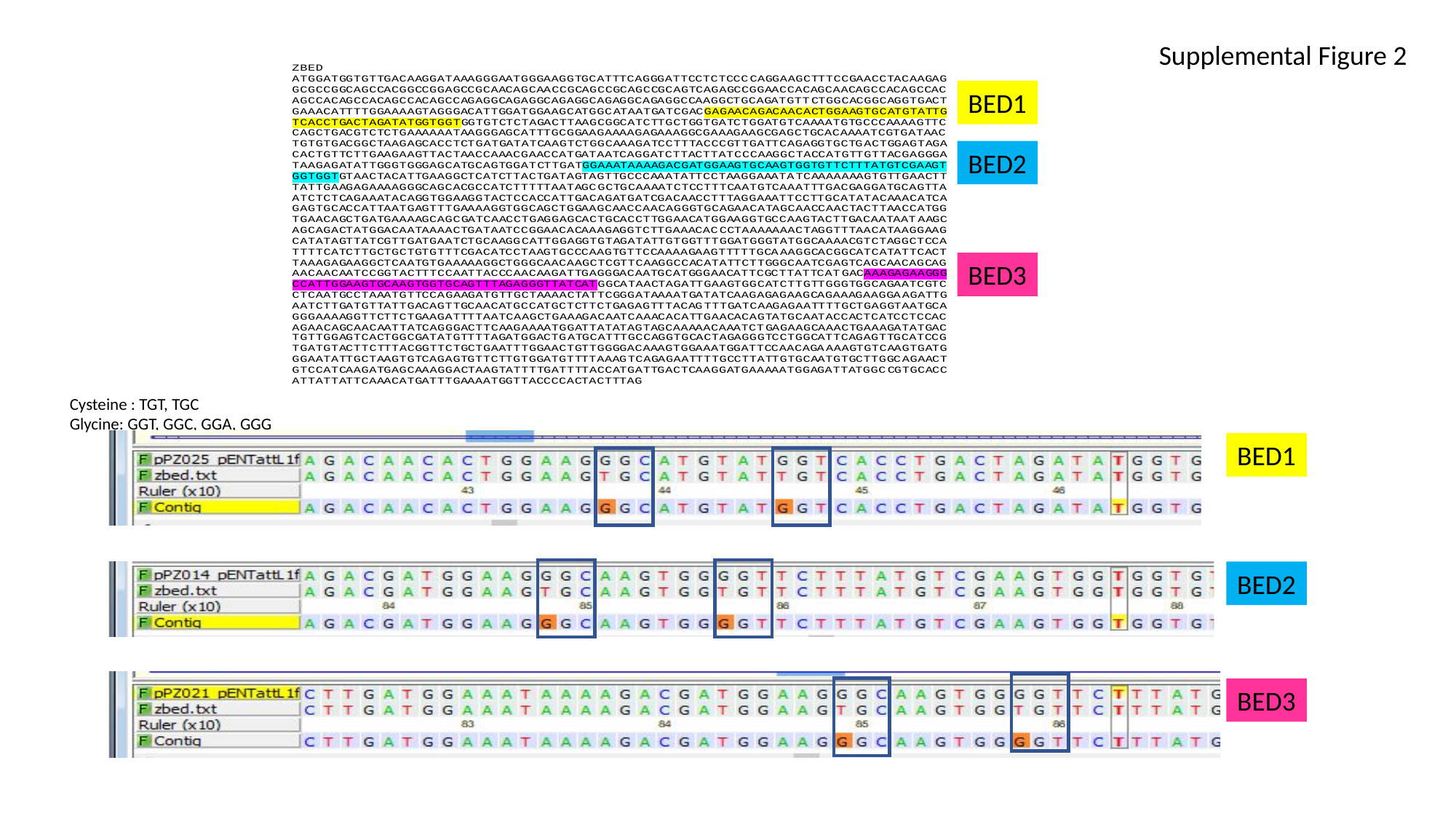

Supplemental Figure 2
BED1
BED2
BED3
Cysteine : TGT, TGC
Glycine: GGT, GGC, GGA, GGG
BED1
BED2
BED3

Supplement: Supplementary Figure 2 — Point mutations in BED domains. Two point-mutations were done in each of the BED domain to substitute the cysteine for glycine, to determine whether the BED domains where responsible for DNA-binding. [file Presentation_2.pptx]

## Slide 1
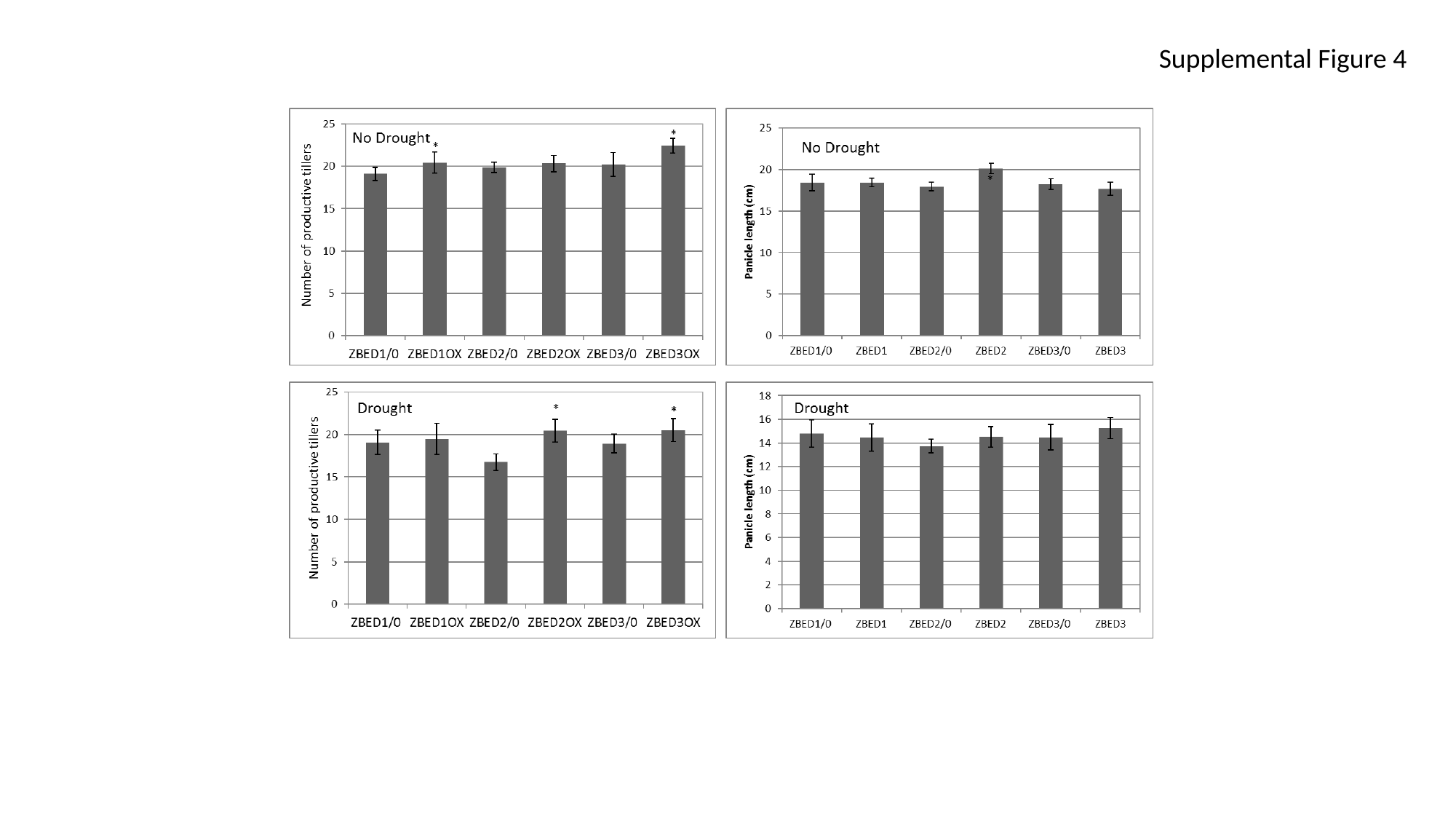

Supplemental Figure 4

Supplement: Supplementary Figure 4 — Evaluation of agronomical traits in rice ZBED overexpressor vs ZBED azygous lines in the field (CIAT, Colombia). Number of productive tillers and panicle length are not significantly different ZBED overexpressor lines under drought stress than in their respective azygous controls (T test p > 0.05). [file Presentation_4.pptx]

## Slide 1
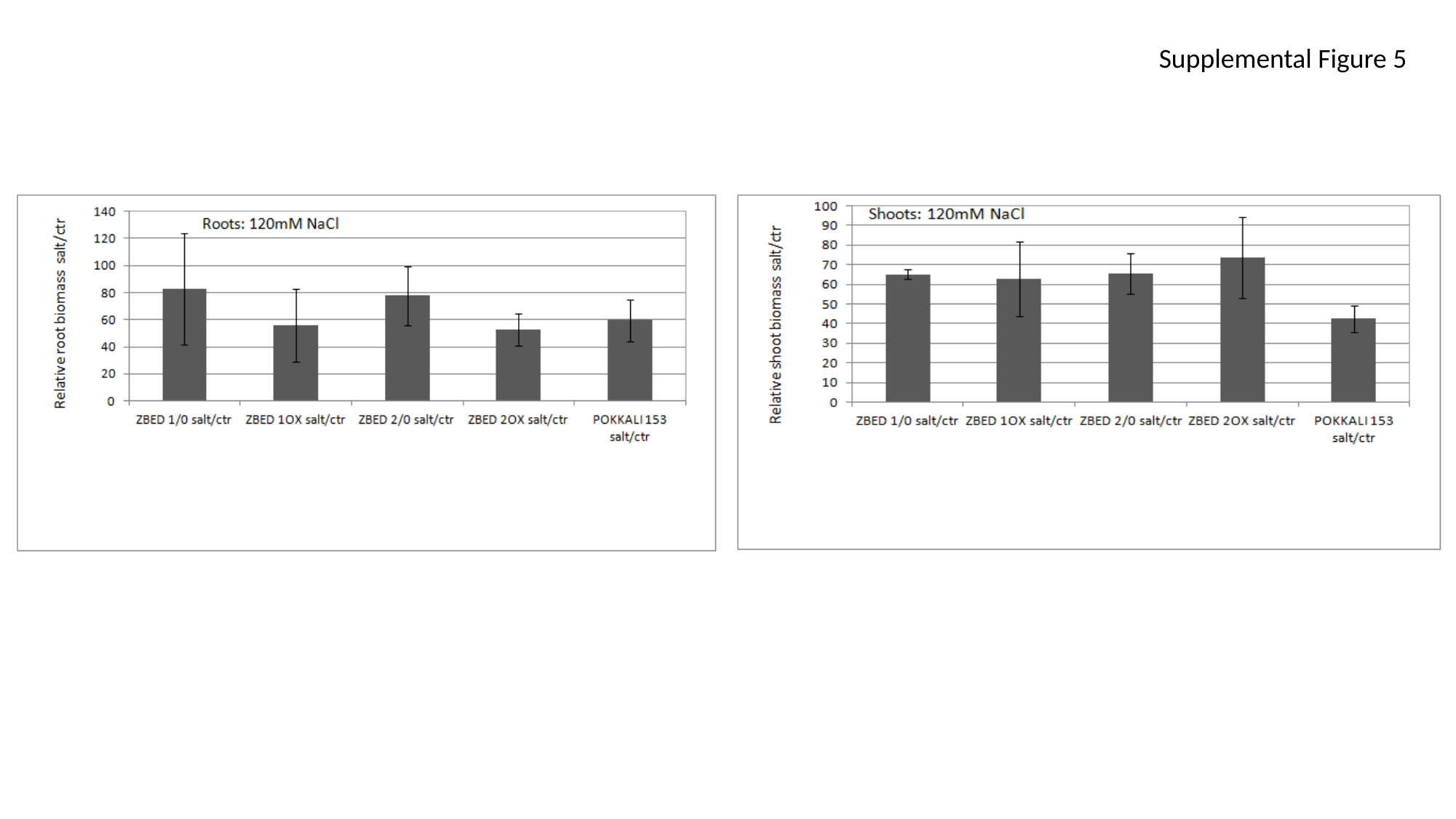

Supplemental Figure 5

Supplement: Supplementary Figure 5 — ZBEDOX and AZY seeds were germinated in MS media, supplemented with 120 mM NaCl to induce salt stress. Pokkali seedlings were used as the resistant control. Relative weight of salt stressed plants over control plants (MS media no-salt added) is shown for root biomass (left) and shoot biomass (right). There is no significant difference between overexpressors or azygous ZBED lines in salt resistance at seedling stage (T test p > 0.05). [file Presentation_5.pptx]

## Slide 1
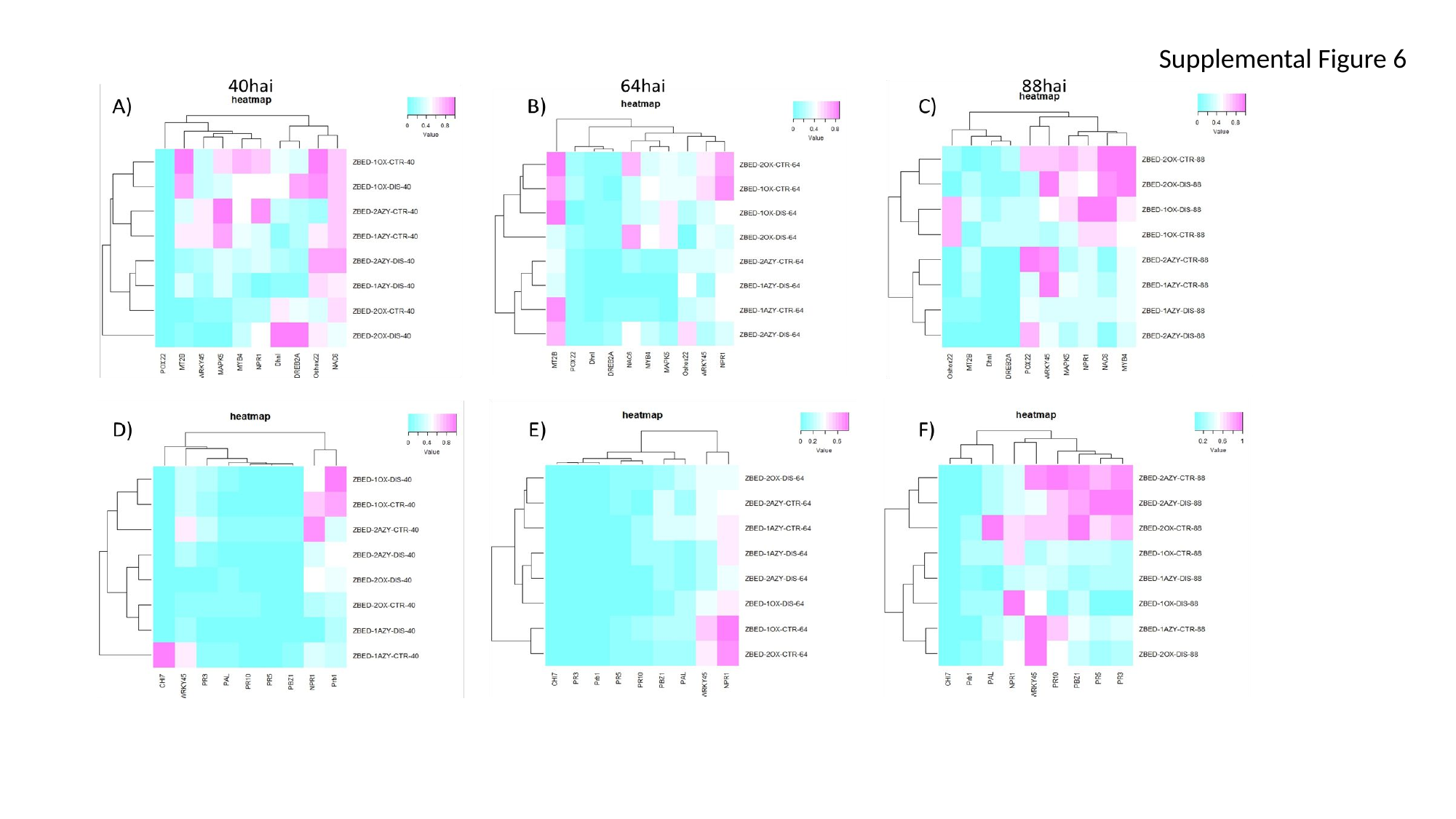

Supplemental Figure 6

Supplement: Supplementary Figure 6 — Heatmap of marker genes for abiotic stress at 40 (A), 64 (B) and 88 (C) h after inoculation (hai) under drought stress (DIS) or no drought (CTR). At 40 hai, abiotic stress markers DREB2A and DhnI show a higher expression in OX plants compared to AZY, while at 64 hai MYB4 and MAPK5 are more expressed in ZBED-OX than ZBED-AZY independently of whether they are inoculated or not. Finally, at 88 hai the expression of MAPK5, NPR1, NAC6, and MYB4 is higher in ZBED-OX plants in both drought and control plants. Heatmap of marker genes for biotic stress at 40, 64, and 88 h after inoculation (hai) (D, E, and F respectively) under drought stress (DIS) or no drought (CTR). Biotic stress markers tested are not differentially expressed in ZBED overexpressor lines 1OX and 2OX compared to their respective azygous lines 1/0 and 2/0 under both drought and M. oryzae infection. [file Presentation_6.pptx]
