# Supplementary material for: The Rice DNA-Binding Protein ZBED Controls Stress Regulators and Maintains Disease Resistance After a Mild Drought
Source: Front Plant Sci. 2020 Aug 18;11:1265. doi: 10.3389/fpls.2020.01265 (PMC7461821; doi:10.3389/fpls.2020.01265)
Supplement: Supplementary Figure 3 — Determining if ZBED is a target for Xanthomonas oryzae effectors. ZBED azygous (ZBED 1/0) and ZBED overexpressor (ZBED 1OX) inoculated with Xanthomonas oryzae pv oryzae (Xoo) PXO99A (A), MaiI (B) and X. oryzae oryzicola (Xoc) BLS256 (D). There is no significant difference (T test p > 0.05) in pathogenicity between ZBED overexpressor lines vs azygous controls with any of the Xoo or Xoc tested. [file Presentation_3.pptx]

## Slide 1
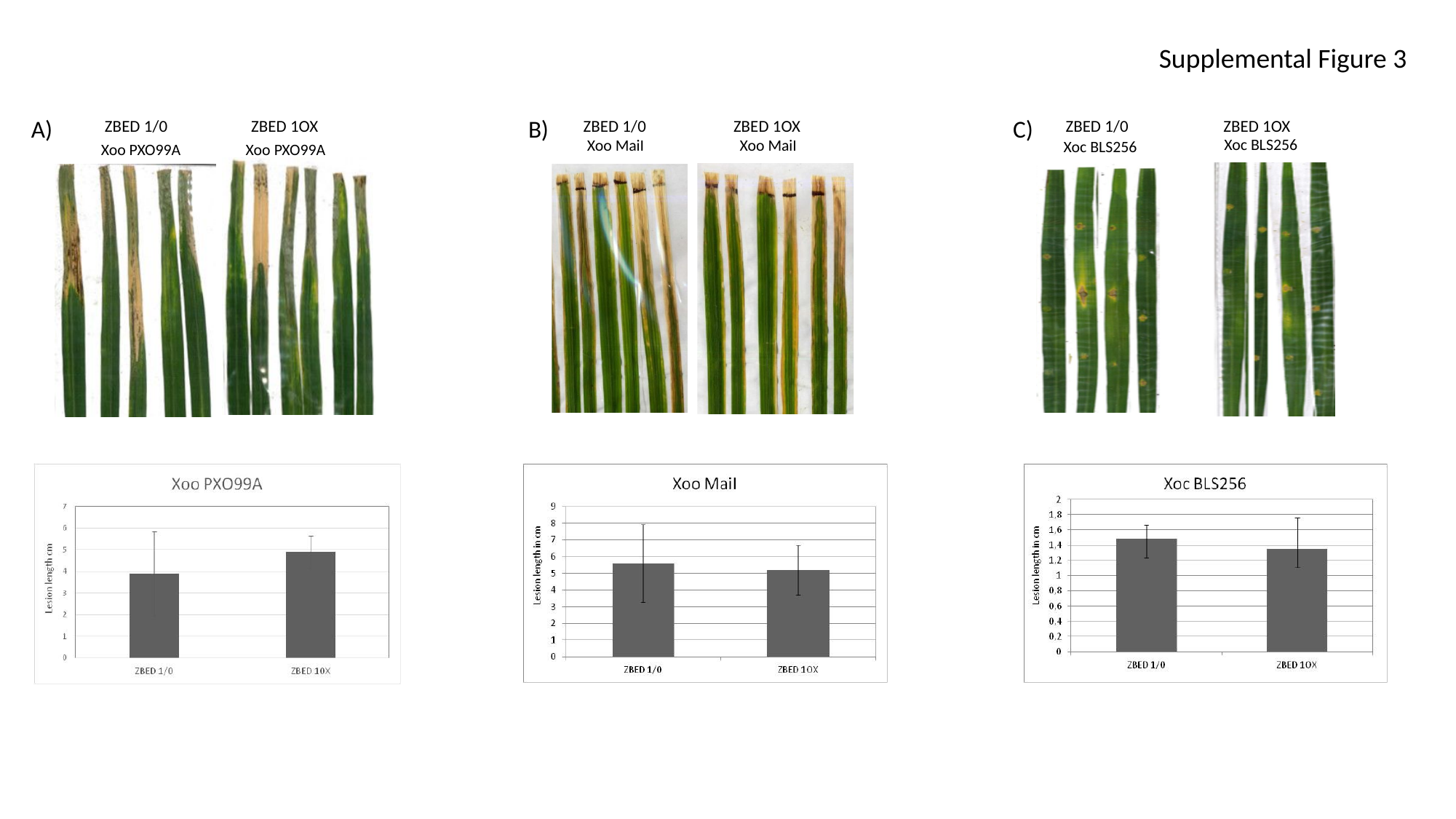

Supplemental Figure 3
A)
B)
C)
ZBED 1/0 ZBED 1OX
Xoc BLS256
Xoc BLS256
ZBED 1/0 ZBED 1OX
Xoo PXO99A Xoo PXO99A
ZBED 1/0 ZBED 1OX
Xoo MaiI
Xoo MaiI
